# Near-Infrared Photoimmunotherapy Using a Small Protein Mimetic for HER2-Overexpressing Breast Cancer

**DOI:** 10.3390/ijms20235835

**Published:** 2019-11-20

**Authors:** Haruka Yamaguchi, Namfon Pantarat, Takamasa Suzuki, Andreas Evdokiou

**Affiliations:** 1 Discipline of Surgery, Breast Cancer Research Unit, Basil Hetzel Institute, University of Adelaide, Adelaide, SA 5011, Australia; haruka.takezawa@adelaide.edu.au (H.Y.); namfon.pantarat@adelaide.edu.au (N.P.); 2 Department of Life Science Dentistry, The Nippon Dental University, Niigata 951-8580, Japan; 3 Department of Oral and Maxillofacial Radiology, The Nippon Dental University School of Life Dentistry at Niigata, Niigata 951-8580, Japan; 4 Faculty of Engineering, Niigata University, Niigata 950-2181, Japan; takamasa@eng.niigata-u.ac.jp

**Keywords:** photoimmunotherapy, NIR-PIT, IR700Dye, Affibody, breast cancer, HER2

## Abstract

Near-infrared photoimmunotherapy (NIR-PIT) is a new and promising cancer therapy based on a monoclonal antibody conjugated to a photosensitizer which is activated by near-infrared light irradiation, causing cell death. We investigated NIR-PIT using a small protein mimetic (6–7 kDa), Affibody molecules, instead of a monoclonal antibody for HER2-overexpressing cancer. Because of its small size, the Affibody has rapid clearance, high imaging contrast, and good tumor penetration. Due to the small size of the Affibodies, which can cross the blood–brain barrier, NIR-PIT using Affibodies has the potential to extend the target cancer of NIR-PIT, including brain metastases. In vitro, NIR-PIT using HER2 Affibody–IR700Dye conjugates induced the selective destruction of HER2-overexpressing breast cancer cells without damage to control cells having low level expression of HER2. HER2-overexpressing cancer cells showed necrotic cell death and their viability maintained at low levels, even 5 days after NIR-PIT. In contrast, treatment with high concentration of HER2 Affibody–IR700Dye conjugate alone or irradiation with high dose of NIR light alone was without effect on cell viability. Affibody and IR700Dye are currently used clinically, and therefore, we would expect the current formulation to be safely and quickly transitioned into clinical trials.

## 1. Introduction

Near-infrared photoimmunotherapy (NIR-PIT) is a new and promising cancer therapy based on the conjugate of monoclonal antibody (mAb) and a photosensitizer (IR700Dye or Indocyanine green). Upon exposure to near-infrared light, the mAb–IR700Dye conjugate is activated, leading to necrotic cell death of cancer cells but without effect on unbound normal cells [1,2,3,4]. NIR-PIT targeting EGFR with a mAb–IR700Dye conjugate is ongoing phase II clinical evaluation for the treatment of head and neck cancer (NCT02422979). This method is applicable to other mAbs and NIR-PIT, with trastuzumab–IR700Dye targeting human epidermal growth factor receptor 2 (HER2) already reported [5,6,7,8,9].

HER2 protein is a 185 kDa transmembrane receptor and belongs to the tyrosine kinase epidermal growth factor receptor family, which promotes cell growth, division, and motility. HER2 overexpression occurs in about 20% of patients with breast cancer and it is generally linked to poor outcomes [10,11,12]. Compared to other subtypes, HER2-positive cancers grow faster due to more HER2 signaling.

Here, we report, for the first time, near-infrared photoimmunotherapy using engineered affinity protein, Affibody molecules, instead of monoclonal antibody to treat HER2-overexpressing breast cancer. Affibody is synthesized based on the immunoglobulin-binding region of staphylococcal protein A to recognize various molecules [13,14,15]. We hypothesize that Affibody represents an appropriate therapeutic agent for NIR-PIT, mainly because its small size (6–7 kDa) and with characteristics that are different from the full antibody, for example, rapid clearance, a good tissue penetration, and binding to a unique epitope of the HER2 receptor [15,16,17]. Additionally, Affibody, due to its small size, can cross the blood–brain barrier [18,19] and it has the potential to provide new possibilities for HER2-overexpressing cancer treatment—including brain metastases. In this study, we aimed to evaluate the efficacy of NIR-PIT using HER2 Affibody-IR700Dye conjugate on HER2-overexpressing breast cancer cells, including cells from brain metastases.

## 2. Results

### 2.1. Human Epidermal Growth Factor Receptor 2 (HER2) Expression

**Figure 1:**
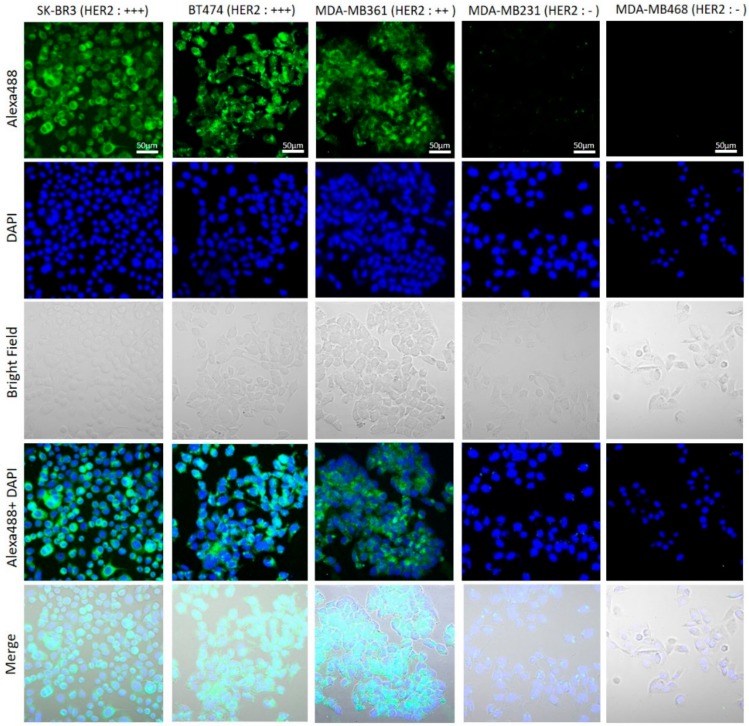
Immunocytochemistry of HER2 protein on breast cancer cell lines (SK-BR3, BT474, MDA-MB361, MDA-MB231, and MDA-MB468). SK-BR3 cells, BT474 cells, and MDA-MB361 cells exhibited stronger fluorescent signals than MDA-MB-231 cells and MDA-MB468 cells in which HER2 expression was virtually undetected. Scale bar: 50 µm.

**Figure 2:**
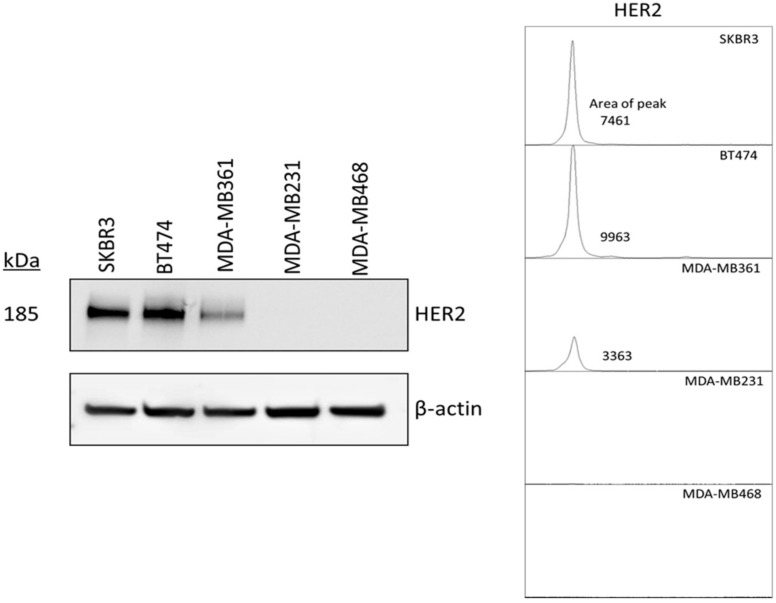
Western blotting of HER2 protein on breast cancer cell lines (SK-BR3, BT474, MDA-MB361, MDA-MB231, and MDA-MB468). β-actin protein expression was assessed as control. A strong band with molecular weight of 185 kDa, corresponding to HER2 protein, was observed in SK-BR3, BT-474, and MDA-MB361. ImageJ analysis for quantifying the protein bands shows area of peaks of each cell line (right panel).

The breast cancer cell lines were first investigated for human epidermal growth factor receptor 2 (HER2) expression by immunocytochemistry (ICC) and Western blotting assay. The results showed that SK-BR3 cells, BT474 cells, and MDA-MB361 cells exhibited stronger fluorescent signals than MDA-MB231 cells and MDA-MB468 cells in which HER2 expression was virtually undetected (Figure 1). In Western blotting assay, a strong band with molecular weight of 185 kDa, corresponding to HER2 protein, was observed in SK-BR3, BT-474, and MDA-MB361, corroborating the ICC results. HER 2 expression was stronger in SK-BR3 and BT-474 cells than MDA-MB361 (Figure 2).

### 2.2. Fluorescence Image of the Cells Bound HER2 Affibody-IR700Dye Conjugate

**Figure 3:**
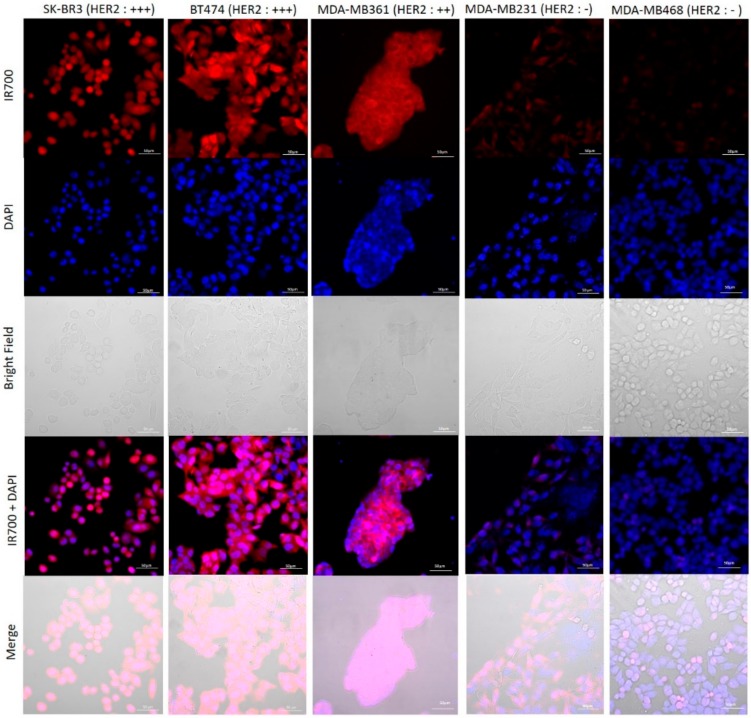
Fluorescence image of the cells bound HER2 Affibody-IR700Dye conjugate. SK-BR3 cells, BT474 cells and MDA-MB361 cells exhibited stronger fluorescent signals of IR700Dye than MDA-MB231 cells and MDA-MB468 cells. Scale bar: 50 µm.

SK-BR3 cells, BT474 cells, and MDA-MB361 cells exhibited stronger fluorescent signals of IR700Dye than MDA-MB231 cells and MDA-MB468 cells, as demonstrated by the addition of HER2-targeted Affibody–IR700Dye conjugate (Figure 3).

### 2.3. Cell Viability after Near-Infrared Photoimmunotherapy (NIR-PIT)

**Figure 4:**
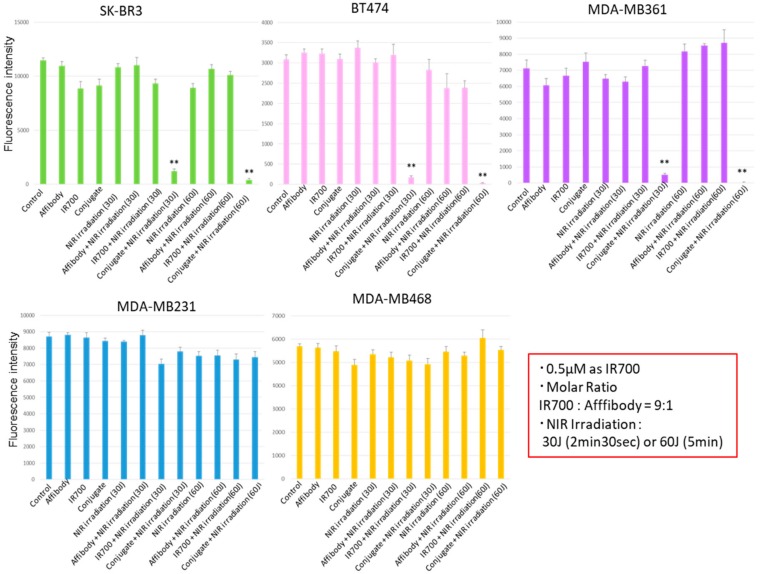
alamarBlue assay showed the cell viability as a fluorescence intensity. The HER2-overexpressing cells (SK-BR3, BT474, MDA-MB361) exposed to near-infrared photoimmunotherapy (NIR-PIT) by HER2 Affibody–IR700Dye conjugate show low fluorescence intensity. In contrast, HER2 low-expressing cells (MDA-MB231, MDA-MB468) maintained strong fluorescence intensity that was at the same level of intensity as the control cells. Data are presented as means ± standard error of the mean (s.e.m.) (*n* = 6; ** *p* < 0.01, Student’s *t*-test).

alamarBlue assay showed the cell viability as a fluorescence intensity. As shown in Figure 4, the HER2-overexpressing cells (SK-BR3, BT474, MDA-MB361) incubated with the HER2 Affibody–IR700Dye conjugate and irradiated with NIR light (30 or 60 J/cm^2^) shows low fluorescence intensity. In contrast, HER2 low-expressing cells (MDA-MB231, MDA-MB468) maintained strong fluorescence intensity that was at the same level of intensity as the control cells, indicating that the NIR-PIT by HER2 Affibody–IR700Dye conjugate dramatically caused selective cell death of HER2-overexpressing breast cancer cells.

**Figure 5:**
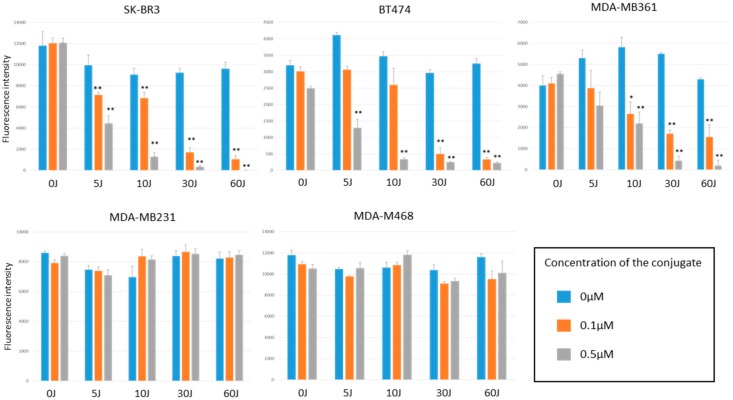
alamarBlue assay showed the cell viability as a fluorescence intensity. Only HER2-overexpressing cells (SK-BR3, BT474, MDA-MB361) exposed to NIR-PIT by HER2 Affibody–IR700Dye conjugate decreased cell viability, which was dependent on the concentration of HER2 Affibody–IR700Dye conjugate and the dose of NIR light irradiation. Data are means ± s.e.m. (*n* = at least 3; * *p* < 0.05; ** *p* < 0.01 vs. non-treatment control, Student′s *t*-test).

As shown in Figure 5, only HER2-overexpressing cells exposed to NIR-PIT by HER2 Affibody–IR700Dye conjugate decreased cell viability, which was dependent on the concentration of HER2 Affibody–IR700Dye conjugate and the dose of NIR light irradiation. Irrespective of high concentration of HER2 Affibody–IR700Dye conjugate and/or high dose of NIR light irradiation, cell viability of HER2 low-expressing cancer cells was maintained at high levels similar to controls.

**Figure 6:**
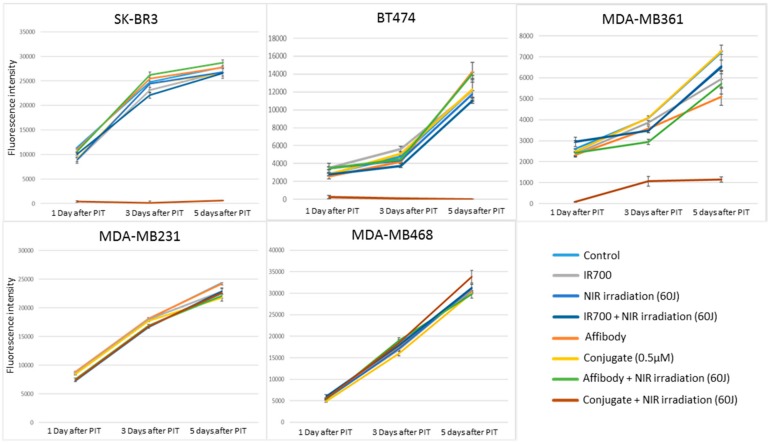
alamarBlue assay after NIR-PIT using HER2 Affibody–IR700Dye conjugate. When cell viability was measured over an extended period of time of 5 days, only the HER2-overexpressing cells (SK-BR3, BT474, MDA-MB361) exposed to NIR irradiation and HER2 Affibody–IR700Dye conjugate were maintained statically low.

When cell viability was measured over an extended period of time of 5 days, only the HER2-overexpressing cells exposed to NIR-PIT by HER2 Affibody–IR700Dye conjugate were maintained statically low, whereas the HER2 low-expressing cancer cells increased their growth exponentially over the 5 days period (Figure 6).

### 2.4. Cell Images Before and after NIR-PIT by HER2 Affibobdy–IR700Dye Conjugate

**Figure 7:**
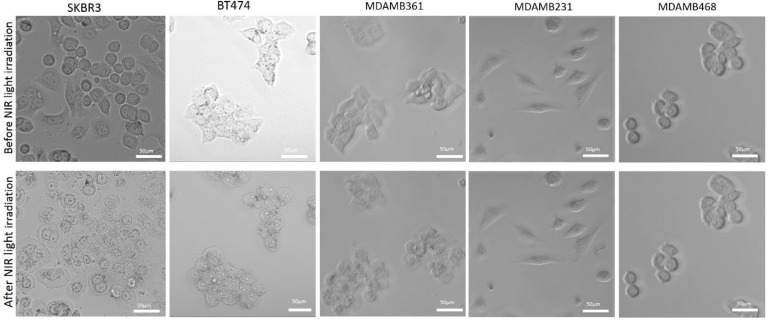
Cell images before and after NIR-PIT by HER2 Affibody–IR700Dye conjugate. Images of cancer cells show that only HER2-overexpressing cells (SK-BR3, BT474, MDA-MB361) displayed morphological evidence of cellular bursting and/or bleb formation, whereas as the morphology of HER2 low-expressing cancer cells (MDA-MB231, MDA-MB468) remained unchanged. Scale bar: 50 µm.

The images of cancer cells show that only HER2-overexpressing cells (SK-BR3, BT474, MDA-MB361) exposed to NIR-PIT by HER2 Affibody–IR700Dye conjugate displayed morphological evidence of cellular bursting and/or bleb formation, whereas the morphology of HER2 low-expressing cancer cells (MDA-MB231, MDA-MB468) remained unchanged (Figure 7).

### 2.5. NIR-PIT by HER2 Affibody–IR700Dye Conjugate Causes Necrotic Cell Death

**Figure 8:**
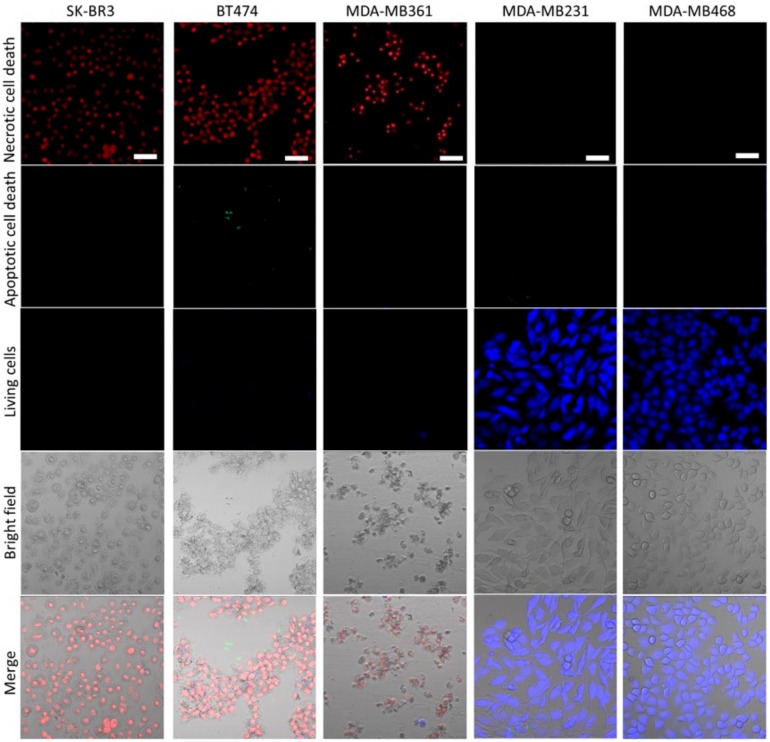
Apoptosis/necrosis assay. Apoptosis/Necrosis Assay Kit stains living cells blue, apoptotic dead cells green, and necrotic dead cells red. HER2-overexpressing cells (SK-BR3, BT474, MDA-MB361) exposed to NIR-PIT by HER2 Affibody–IR700Dye conjugate show red signal, whereas HER2 low-expressing cells (MDA-MB231, MDA-MB468) stained blue. Scale bar: 50 µm.

Apoptosis/Necrosis Assay Kit stains living cells blue, apoptotic dead cells green, and necrotic dead cells red. As shown in Figure 8, HER2-overexpressing cells exposed to NIR-PIT by HER2 Affibody–IR700Dye conjugate show red signal, whereas HER2 low-expressing cells stained blue, indicating that the mode of death by NIR-PIT for HER2-overexpressing cells is selectively necrotic.

### 2.6. Calcein AM and Propidium Iodide (PI) Staining for NIR-PIT-Treated Cells

**Figure 9:**
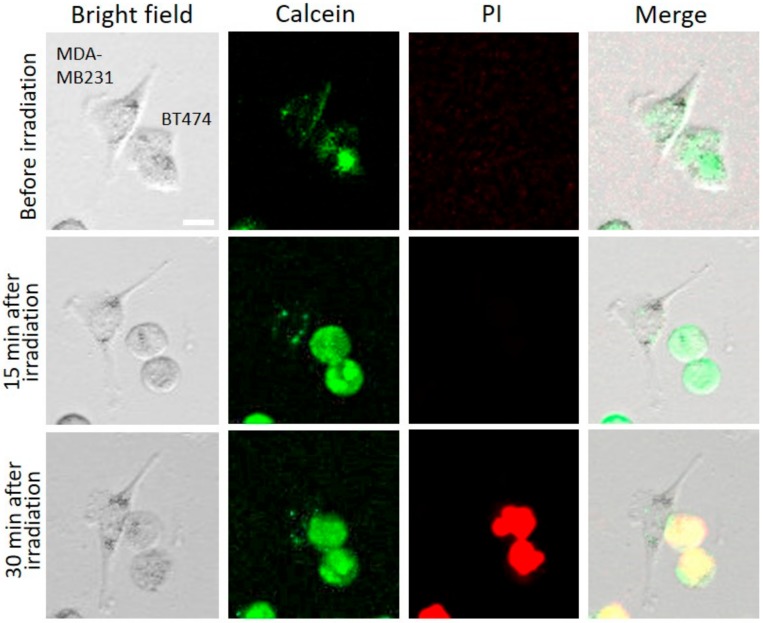
Cell images of calcein and propidium iodide (PI) analysis before NIR irradiation, 15 min after NIR irradiation, and 30 min after NIR light irradiation. HER2-overexpressing cells (BT474) and HER2 low-expressing cells (MDA-MB231) were stained by calcein before NIR light irradiation. While BT474 cells were swelling, green fluorescent remained intracellular. The BT474 cells that got big cell membrane damage due to bursting were stained by PI, whereas MDA-MB231 were intact. Scale Bar: 20 µm.

Calcein AM is used as green fluorescent staining for the living cells, and with PI, it is possible to stain the dead cells by red fluorescence. The permeability of NIR-PIT-treated cells were determined by PI staining, followed by fluorescence microscopy, based on the principle that damaged cells become fluorescent as PI dye begins to internalize and bind to nucleic acids. In Figure 9, HER2-overexpressing cells (BT474) and HER2 low-expressing cells (MDA-MB231) were mixed and treated by NIR-PIT using HER2 Affibody–IR700Dye conjugate. Before NIR light irradiation, both cell types (BT474, MDA-MB231) were stained by calcein. Fifteen minutes after NIR light irradiation, only BT474 cells showed evidence of swelling, whereas control cells (MDA-MB231) were intact. While BT474 cells were swelling, green fluorescent (calcein) remained intracellular. Thirty minutes after NIR light irradiation, the membranes of BT474 cells were damaged due to bursting and were therefore stained by PI. These results suggest that NIR-PIT by HER2 Affibody–IR700Dye conjugate induced cell-membrane damage of only targeted cells.

## 3. Discussion

To determine the effect of near-infrared photoimmunotherapy (NIR-PIT) using HER2 Affibody–IR700Dye conjugate, we performed a range of in vitro analyses. Our immunocytochemistry (ICC) and Western blot analysis demonstrated strong expression of HER2 protein by SK-BR3, BT474, and MDA-MB361 cancer cells compared to MDA-MB231 and MDA-MB468 cancer cells (Figure 1 and Figure 2). These results are in line with those reported by others in the literature [20,21,22]. Fluorescence imaging of the cells bound to HER2 Affibody–IR700Dye conjugate show a similar fluorescence intensity of IR700Dye to green signal by ICC (Figure 3), suggesting that the HER2 Affibody–IR700Dye conjugate bound to HER2 protein on cancer cells was highly specific. NIR-PIT by HER2 Affibody–IR700Dye conjugate caused selective cell death of HER2-overexpressing breast cancer cells (Figure 4, Figure 5 and Figure 6). While the expression of HER2 protein on MDA-MB361 cells was lower than the other HER2-overexpressing cancer cells (Figure 2) [21], long-term cell survival was, nonetheless, maintained at low levels, albeit a slight recovery evident at 5 days when HER2-overexpressing cells were treated with NIR-PIT by the Affibody–IR700Dye conjugate (Figure 6). Collectively, our results clearly indicate that the effect of NIR-PIT cells in vitro is well correlated with the level of HER2 protein expression on targeted cells. In Figure 5, the cell viability decreased as exposure to NIR light dose increased or the concentration of the conjugate increased, indicating that MDA-MB361 cells need higher NIR light dose or higher concentration of the conjugate to ensure complete cell death.

Our results show that NIR light irradiation induces necrotic cell death of only HER2-overexpressing cancer cells incubated with HER2 Affibody–IR700Dye conjugate, without any damage to HER2 low-expressing cancer cells (Figure 8). According to Sato et al., the NIR-PIT using mAb induces physical changes of the conjugate that is bound on the surface of the target cells, exerting physical stress within the cellular membrane, leading to an overall increase in transmembrane water flow that eventually leads to cell bursting and necrotic cell death [3]. We show here that (Figure 7, Figure 8 and Figure 9), after NIR light irradiation, HER2-overexpressing cells incubated with the HER2 Affibody conjugate displayed morphological changes of swelling and bursting. In Figure 9, while HER2-overexpressing cells (BT474) were swelling, green calcein fluorescence remained intracellular and was gradually positively stained with propidium iodide (PI). Ogawa et al. described that the cell-membrane damage induced by NIR-PIT initially induces osmotic damage to the membrane, permitting the passage of water but not larger or charged molecules. After further cellular membrane rupture, larger molecules can flow into and out of cells [23]. We speculate that NIR-PIT using HER2 Affibody–IR700Dye conjugate causes a similar physical stress against the cell membrane of targeted cells as NIR-PIT using mAb.

NIR-PIT using HER2 Affibody–IR700Dye conjugate has added potential for clinical use. Firstly, Affibody (6–7 kDa) has a rapid clearance and a good tumor penetration [15,16]. Secondly, HER2 Affibody targets a unique epitope of the HER2 receptor and does not compete with Trastuzumab or Pertuzumab (HER2 monoclonal antibody, molecular targeted drug) [17,18,24]. This implies that NIR-PIT using HER2 Affibody–IR700Dye conjugate may be available to treat HER2-overexpressing cancers, regardless of any other ongoing molecular-targeted therapy using Trastuzumab or Pertuzumab. Moreover, it can be used to amplify the effect of NIR-PIT by combination therapy, targeting two epitopes using both mAb conjugate and the HER2 Affibody–IR700Dye conjugate [9]. Additionally, HER2 Affibody binds the HER2 protein on the membrane of Trastuzumab-resistant HER2-overexpressing cells, suggesting the potential of NIR-PIT using HER2 Affibody–IR700Dye conjugate to treat Trastuzumab-resistant cancer [20]. Thirdly, because of their small size, Affibody conjugates can cross the blood–brain barrier and therefore, can be used to clearly image brain metastases or glioma [18,19,25,26]. Hua Jing et al. reported NIR-PIT for glioma using mAb–IR700Dye conjugate [27]. However, Burley et al. described that the relatively large molecular size of mAbs may limit their extravasation into the glioblastoma (GBM) tumor, especially in areas with only partial blood–brain barrier disruption. They reported EGFR Affibody–IR700Dye conjugate specific uptake in vitro enabled imaging of EGFR expression in an orthotopic brain tumor model, and an NIR-PIT study in vivo demonstrated therapeutic efficacy of the conjugate in a subcutaneous glioma xenograft model [19]. Our findings that the MDA-MB361 cell line, which is derived from a brain metastasis of breast cancer, can be effectively treated by NIR-PIT using HER2 Affibody–IR700Dye conjugate may extends the therapeutic prospect of this approach for brain metastases. Taken together, HER2 Affibody–IR700Dye conjugate represents an approximately designed therapeutic agent with specific characteristics for NIR-PIT against HER2-overexpressing cancer. When NIR-PIT by the HER2 Affibody–IR700Dye conjugate is contemplated for brain metastases of HER2-overexpressing breast cancer, HER2 status can be examined by imaging [18,28], and NIR light can be irradiated directly during surgery or delivered by an optical fiber diffuser providing the NIR light directly to the tumor [29,30]. 36.2% brain metastases from breast cancer patients overexpressed HER2, and the brain metastases indicated an enrichment in the frequency of tumor HER2 overexpression at this metastatic site [10,31,32]. NIR-PIT using HER2 Affibody–IR700Dye conjugate has a real therapeutic potential for brain metastases of HER2-overexpressing cancers. Lastly, NIR-PIT using mAb to target HER2 protein is already reported for ovarian cancer, gastric cancer, bladder cancer, as well as breast cancer [5,6,7,29,33]. HER2 Affibody–IR700Dye conjugate can also target these tumors. Importantly, Affibody and IR700Dye are currently used clinically; therefore, we would expect the current formulation to be safely and quickly translated into clinical practice (https://clinicaltrials.gov/ct2/show/NCT02422979), [18,34,35,36,37].

This is the first ever report of NIR-PIT using HER2 Affibody–IR700Dye conjugate for breast cancer. Although further studies are required to examine these the full therapeutic potential of this approach, NIR-PIT using HER2 Affibody–IR700Dye conjugate is expected to improve the present NIR-PIT.

## 4. Materials and Methods

### 4.1. Cell Culture

The human breast cancer cell lines SK-BR3, BT474, MDA-MB361, MDA-MB231, and MDA-MB468 were obtained from the American Type Culture Collection (ATCC^®^, Manassas, VA, USA). SK-BR3 cell line was cultured in McCoy’s 5A medium (GIBCO^®^, Life Technologies, Carlsbad, CA, USA) supplemented with 10% fetal bovine serum (FBS; GIBCO^®^, Life Technologies, Carlsbad, CA, USA) and 1% penicillin–streptomycin (Invitrogen, Life Technologies, Carlsbad, CA, USA). Other cell lines were cultured in DMEM medium (GIBCO^®^, Life Technologies, Carlsbad, CA, USA) supplemented with 10% fetal bovine serum and 1% penicillin–streptomycin. All cell lines were maintained in a humidified environment containing 5% CO_2_ at 37 °C. The medium was changed every other day.

### 4.2. Immunocytochemistry (ICC)

In total, 1 × 10^5^ HER2-overexpressing breast cancer cells (SK-BR3, BT474, MDA-MB361) and HER2 low-expressing breast cancer cells (MDA-MB231, MDA-MB468) were seeded on coverslips at the bottoms of wells in a 24 well-plate. Cells were fixed in 4% paraformaldehyde for 15 min, washed with phosphate-buffered saline (PBS) twice, and non-specific sites were blocked with 3% bovine serum albumin (BSA) in PBS for 30 min at room temperature. Cells were incubated with anti-HER2 antibody (HER2/ErbB2 (D8F12) XP^TM^ Rabbit mAb, Cell Signaling technology, Danvers, MA, USA) overnight at 4 °C, followed by incubation with the appropriate Alexa Fluor 488 secondary antibody (1:1000, Anti-Rabbit IgG Fab2, Alexa Fluor (R) 488, Cell Signaling technology) for 1 h at room temperature. The cover slips were added the mounting medium with DAPI for 10 min prior to imaging by fluorescence microscope (LSM 700 confocal, ZEISS, Oberkochen, Germany).

### 4.3. Western Blot Analysis

The general procedure for the Western blot analysis was performed as follows. Cells were washed with ice-cold PBS and added 50 µl of modified RIPA buffer (Thermo Fisher Scientific, Waltham, MA, USA), containing protease inhibitor tablet (cOmplete™, Mini Protease Inhibitor Cocktail, Roche, Mannheim, Germany), 1% EDTA, and 1% protease/phosphatase inhibitor (Thermo Fisher Scientific) per well of the 6-well plate. The cells were scraped from wells, collected, and centrifuged at high speed at 4 °C for 4 min. Supernatant was transferred to fresh tubes and protein concentration was determined by using Pierce BCA Protein Assay (Thermo Fisher Scientific) as the manufacturing protocol described. The samples were then heated for 5 min at 100 °C and equal amounts of proteins (50 µg) were subjected to SDS-PAGE. The proteins were transferred to a polyvinylidene fluoride (PVDF) membrane. After blocking with 5% non-fat milk, the membrane was incubated with primary antibody (HER2/ErbB2 (D8F12) XP^TM^ Rabbit mAb, Cell Signaling technology) at 4 °C overnight. Thereafter, membrane was incubated with secondary antibodies (Anti-Rabbit Immunoglobulins/HRP P0448, Dako, Via Real Carpinteria, CA, USA). As a loading control, the membrane was also subjected to immunoblotting using β-actin mAb (Monoclonal Anti-β-Actin, AC-15, A5441, Sigma-Aldrich, St. Louis, MO, USA) as primary antibody and anti-mouse IgG antibody (P0161, Rabbit Anti-Mouse Immunoglobulins/HRP, Dako, CA, USA) as secondary antibody. The immunoreactive bands were visualized with a chemiluminescent using the ECL Western blotting detection reagent. Image Quant LAS-4000 (GE Healthcare, Chicago, IL, USA) was used to visually assess and ImageJ (NIH, Bethesda, MD, USA and LOCI, Madison, WI, USA) was used for quantifying the protein bands.

### 4.4. HER2 Affibody–IR700Dye Conjugate

HER2 affibody (Affibody AB, Solna, Sweden) was dissolved in PBS to a final concentration of 1 mg/mL and was added dithiothreitol (DTT) to a final concentration of 20 mM at > pH 7.5. After incubation at room temperature for 2 h, excess DTT was removed from the conjugate by passage through an NAP5 column (GE Healthcare, IL, USA). Next, the HER2 Affibody was incubated with a 9-fold molar excess of IRDye700DX–maleimide (MW: 1979.23, LI-COR Biosciences, Lincoln, NE, USA) for 2 h at 37 °C. After conjugation, the solution was applied to protein desalting spin columns (Thermo Fisher Scientific) and centrifuged at 1500× *g* for 2 min twice to purify the sample.

### 4.5. Confocal Microscopy Imaging of HER2 Affibody–IR700Dye Conjugate Staining

HER2-overexpressing breast cancer cells (SK-BR3, BT474, MDA-MB361) and HER2 low-expressing breast cancer cells (MDA-MB231, MDA-MB468) were seeded on the coverslips at the bottoms of wells in a 24-well plate. To test the specificity of the conjugate binding, HER2 Affibody–IR700Dye conjugate (1 µM) was added to the media and the cells were incubated for 30 min at 37 °C. After washing the cells with PBS, the coverslips were put on a glass slide, and the cells were examined using fluorescence microscopy (LSM confocal, ZEISS, Oberkochen, Germany).

### 4.6. Cell Viability Assay

The cell viability was determined using fluorescence intensity of an alamarBlue assay. Briefly, cells were seeded at 1 × 10^4^/well in flat-bottom 96-well culture plates and allowed to grow for 24 h, followed by incubation with Affibody or IR700Dye only or HER2 Affibody–IR700Dye conjugate (0–0.5 µM) for 2 h at 37 °C. After washing the cells twice with PBS, near-infrared light (0–60 J/cm^2^) was irradiated from the bottom of wells. After near-infrared (NIR) light irradiation, the cells were incubated with alamarBlue solution (10 µL/100 µL in medium) for 2 h and the fluorescence intensity was measured at 540–570/580–610 nm using a micro plate reader (BMG FLUO star OPTIMA, BMG Labtech, Offenburg, Germany). The cell viabilities were followed for 5 days after NIR light irradiation. The results of representative experiments are presented as the mean ± standard error of the mean (s.e.m.) (* *p* < 0.05; ** *p* < 0.01 vs. non-treatment control), which were performed at least three wells per sample and repeated more than three times. Student’s *t*-test was used for analyses.

### 4.7. Cell Images Before and after Near-Infrared (NIR) Light Irradiation

Cells were seeded at 1 × 10^4^/well in flat-bottom 96-well culture plates and allowed to grow for 24 h, followed by incubation with HER2 Affibody–IR700Dye conjugate (0.5 µM) for 2 h at 37 °C. After washing the cells twice with PBS, near-infrared light (60 J/cm^2^) was irradiated from the bottom of wells. The images of cells were taken by microscope (LSM confocal, ZEISS, Oberkochen, Germany) before and after NIR irradiation.

### 4.8. Cell Apoptosis/Necrotic Assay

Cells (SK-BR3, BT474, MDA-MB361, MDA-MB231, MDA-MB468) were seeded at 1 × 10^4^/well in flat-bottom 96-well culture plates and allowed to grow for 24 h, followed by incubation with HER2 Affibody–IR700Dye conjugate (0.5 µM) for 2 h at 37 °C. After washing the cells twice with PBS, near-infrared (NIR) light (60 J/cm^2^) was irradiated from the bottom of wells. Then, apoptosis or necrosis of the cells was determined using the Apoptosis/Necrosis Assay Kit (ab176749, Abcam, Cambridge, UK) as the manufacturing protocol described.

### 4.9. Calcein AM/Propidium Iodide (PI) Staining of Mixed Cell Lines

HER2-overexpressing cells (BT474) and HER2 low-expressing cells (MDA-MB231) were mixed and seeded in 96-well plate at 1 × 10^4^ of each cell line/well, then allowed to adhere overnight. The cells were incubated with HER2 Affibody–IR700Dye conjugate (0.5 µM) for 2 h at 37 °C. After washing with PBS twice, the cells were added calcein AM (Invitrogen, MA, USA) and propidium iodide (Invitrogen, Waltham, MA, USA) at 3 µM and 2.5 µM final concentration, respectively. Then, NIR light (30 J/cm^2^) was irradiated from the bottom of wells. The images of the cells were acquired before NIR light irradiation and immediately after irradiation, every 1 min for 1 h using a fluorescence microscope (LSM confocal, ZEISS, Oberkochen, Germany). The cells were maintained in a humidified atmosphere of 5% CO_2_ at 37 °C during the imaging.

### 4.10. Near-Infrared Photoimmunotherapy (NIR-PIT) Illuminator

**Figure 10:**
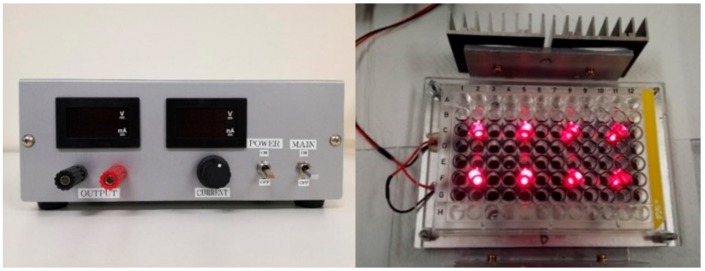
Our own designed NIR-PIT illuminator was constructed by 8 light-emitting diodes (LED: SMBB690D-1100-02 × 8, EPITEX, Inc., Kyoto, Japan), whose peak wavelength of emission is 690 nm. The power density of the LEDs is controllable from 0 to 600 mA (0–244.86 mW/cm^2^).

Our own designed NIR-PIT illuminator (Figure 10) was constructed of 8 light-emitting diodes (LED: SMBB690D-1100-02 × 8, EPITEX, Inc., Kyoto, Japan), whose peak wavelength of emission is 690 nm. Its narrow directivity allows irradiation of cells with concentrated single direction. The power density of the LEDs is controllable from 0 to 600 mA (0–244.86 mW/cm^2^). In this study, the power density was set to 200 mW/cm^2^ at 500 mA for all cellular experiments of NIR-PIT. The power density passing through the bottom of the well was measured by an optical power meter, which was made by combining a photo diode detector (PH100-Si-HA, Gentec Electro-Optics, Inc., St-Jean-Baptiste, Quebec, Canada) and a touchscreen display device (MAESTRO, Gentec Electro-Optics, Inc., Quebec, Canada).

## 5. Conclusions

NIR-PIT using HER2 Affibody–IR700Dye conjugate represents a new and promising therapeutic approach for the treatment of breast cancer.

## Abbreviations

HER2	Human epidermal growth factor type 2
EGFR	Epidermal growth factor receptor
NIR-PIT	Near-infrared photoimmunotherapy
mAb	Monoclonal antibody
ICC	Immunocytochemistry
PI	Propidium iodide
